# The role of chloramines in treatment of diabetic foot ulcers: an exploratory multicentre randomised controlled trial

**DOI:** 10.1186/s40842-016-0026-8

**Published:** 2016-03-30

**Authors:** Karin Bergqvist, Ulrica Almhöjd, Irene Herrmann, Björn Eliasson

**Affiliations:** 1 Bergsjöns Vårdcentral, Göteborg, Sweden; 2 Department of Cariology, Institute of Odontology, Sahlgrenska Academy, University of Gothenburg, Gothenburg, Sweden; 3 RLS Global AB, Gothenburg, Sweden; 4 Department of Medicine, University of Gothenburg, Sahlgrenska University Hospital, Gothenburg, Sweden

**Keywords:** Diabetes mellitus, Diabetic foot, Foot ulcer, Chloramines

## Abstract

**Background:**

Chronic foot ulcers in diabetes are serious, costly and frequently difficult to heal. Recent guidelines conclude that new dressings and treatments generally fail to show superiority compared with standard of care. Several mechanisms are probably responsible for impaired healing of chronic foot ulcers, including inflammation and infection. Chloramines have presumed antiseptic and antibacterial properties, and have shown to be a useful treatment in odontology.

**Methods:**

In an explorative open randomised controlled multi-centre study, we compared chloramine-based treatment with current standard of care for 12 weeks and follow-up for 24 weeks. Seventeen patients in each group, mean age about 70, duration of diabetes > 20 years and foot ulcers about 1.5 years, completed the 12 weeks study.

**Results:**

After 5 weeks, the difference between the groups in relative reduction in ulcer area was statistically significant (p=0.016). Absolute change in ulcer area was first statistically significant within the chloraminetreated group after 2 weeks (p=0.026), after 8 weeks in the control group (p=0.0023), with significant difference between groups after 5 weeks (p=0.024). The approximate relative decrease per week was 19.4% (95%CI 12.2, 26.0; p<0.0001) in the chloramine-treated group and 11.7% (95%CI 6.4, 16.7; p<0.0001; between-group difference p=0.083). After 9 weeks 7 patients had healed in the chloraminetreated group, but only one in the control group (p=0.039). There were no statistically significant differences in wound healing at 12 or 24 weeks, and no marked differences in signs of infection, pain, quality of life (EQ-5D), or incidence of adverse events.

**Conclusions:**

Chloramine-based treatment seems to be efficacious, particularly in the early phase of the care of infected diabetic foot ulcers. It is safe and easy to use, and could prove to be a valuable addition in the treatment arsenal, providing non-surgical debridement. Future studies will evaluate its role in wound care.

## Background

Non-healing foot ulcer is a serious and costly complication of diabetes mellitus [1]. The pathophysiology is multifactorial, including neuropathy, microvascular and macrovascular angiopathy, foot deformities, impaired wound healing and infections [2]. Thus, management of diabetes foot ulcers has to include multiple modalities, such as effective offloading, improvement of circulation, wound bed preparation and removal of dead tissue, and treatment of infection. However, with few exceptions, new treatment options have usually failed to show superior effectiveness compared with modern standards of care [3, 4].

Several mechanisms are probably responsible for impaired healing of chronic foot ulcers, such as an inflammatory state, including ineffective neoangiogenesis, fibroblast infiltration, granulation, and re-epithelialization [5]. Bacteria colonizing diabetic foot ulcers in complex biofilms have also been suggested to contribute to its treatment-resistant state [5–8]. Organic chloramines have multiple effects, which could be beneficial in wound care. They have presumed antiseptic and antibacterial properties [9, 10], and have also been shown to degrade toxins and biofilms [11, 12]. In odontology chloramines facilitate effective chemo-mechanical cleansing and debridement while preserving healthy tissue [13–16].

The aim of this study was to assess whether a chloramine preparation can be used in conjunction with weekly dressings to improve healing of diabetic foot ulcers. A product used for periodontitis (Perisolv™, RLS Global AB, Gothenburg, Sweden) was modified and used in an explorative open randomized and controlled study, compared with current standard treatment.

## Methods

### Patients and baseline characteristics

The Ethics Review Board of the University of Gothenburg and the Swedish Medical Products Agency approved this study. We recruited and treated 39 patients with diabetes and foot ulcers at four centres in the Gothenburg area. The patients were randomized in blocks of four to standard treatment or treatment with chloramine.

We used these inclusion criteria: type 1 or type 2 diabetes, age 18 years or older, and an infected foot ulcer for more than 4 weeks. The presence of infection was defined as two or more markers of inflammation (local warmth, erythema, local swelling or induration around the ulcer, local tenderness or pain), purulent discharge, i.e. mild or moderate infection, as defined by the Infectious Diseases Society of America [17]. Initially a minimum ulcer size of 300 mm^2^ was required, but after 10 months the minimal ulcer size was changed to 25 mm^2^ due to slow recruitment (after a survey evaluating ulcer size in 60 consecutive patients, followed by formal approvals). We excluded patients with end-stage renal disease, impaired blood circulation (toe arterial blood pressure < 30 mmHg, measured according to Johansson et al. [18], or in need of vascular intervention, or a vascular intervention performed less than three months before the study, a history of kidney or pancreas transplant, treatment with cortisone at higher doses than 60 mg daily, chemotherapy or any immune-modulating agents during the past year, other identified conditions in the area of the ulcer (e.g. cancer), or generally poor condition at risk of requiring hospitalisation. Although previous use of biological dressings was not an exclusion criterion, no patients were previously treated with this modality. We also did not include patients with a severe infection (poor general condition or metabolic disorders).

At the screening appointment, the following characteristics were documented: age, sex, type and duration of diabetes, of diabetes, history of myocardial infarction or stroke, HbA1c, duration and position of the ulcer. The area of the ulcer was calculated (rectangular: length × width; circular: radius × radius × pi (3.14); triangular: base × height/2). Sensory neuropathy was determined using a tuning fork (128 Hz).

### Treatments

Nurses and podiatrists performed cleansing and debridement of the ulcers in both groups at least once weekly for 12 weeks, but were treated (if needed) and followed for 24 weeks. In patients randomized to chloramine treatment, we applied a preparation containing sodium hypochlorite and amino acids, which are converted to chloramines by mixing the two components immediately prior to the treatment [19]. The formed chloramines are mild oxidising agents, which can transfer chlorine to other substrates, regenerating the parent amine in the process [20]. The gel was applied on the ulcer during treatment sessions once a week. Debridement was performed without sharp instruments, with the gel left on the ulcer surface to give antibacterial protection for the exposed tissue. This procedure lasted five minutes and was done twice. The ulcer was then rinsed with water, leaving no substance that could interfere with healing. Finally, the ulcer was dressed with a non-adhesive protective dressing and self-adhesive fabric. If needed, a new dressing could be applied between the weekly appointments.

In the patients randomized to standard treatment, the ulcer was cleansed and debrided according to guidelines presented by International Working Group of Diabetic Foot once weekly (or more often if needed) [21]. The ulcer was dressed with foam, hydrocolloid or alginate dressings, keeping a moist wound environment. In a few cases an adjusted antiseptic agent, silver or PHMB, was also used.

A photo was taken every week after the treatment, with a ruler (millimetres) in the photo with the patient ID number and date. The area of the ulcer was subsequently measured from the photos by an independent and blinded observer. We also evaluated pain (visual-analogue scale) and quality of life (EQ-5D).

All patients were given standard of care advice concerning the treatment of diabetes and risk factors. Oral antibiotic treatment was offered in situations with signs of significant infection, particularly affecting underlying tissue or bone. Appropriate off-loading was considered and emphasized in all patients, i.e. individually tested orthopaedic shoes and inserts, use of removable cast walker boots (e.g., Aircast) or even wheelchairs. If needed, patients were referred for peripheral vascular examinations or intervention.

### Statistical analyses

Ulcer sizes were measured once weekly for 12 weeks, and a final evaluation was carried out after 24 weeks to document the status of the ulcer (healed or not), time to healing and occurrence of adverse effects. In order to detect a difference of 300 mm^2^ in the primary efficacy variable (absolute change in area of the ulcers from the baseline visit to week 12 between the chloramine treatment group and the standard treatment group with two-sided t-test with a power of 90 % and significance level of 0.05 %) at least 18 patients were needed in each group. We assumed equal standard deviation (SD) of 266 mm^2^ in both groups. In a comparable study the SD was estimated to 267 mm^2^ in the active group and 86 mm^2^ in the control group [22]. In order to adjust for loss to follow-up and early discontinuation, 20 patients in each group, 40 in total, needed to be randomized.

For descriptive purposes, means, standard deviations (SD), medians, minimum and maximum values are given for continuous variables and number and percentages for categorical variables. Non-parametric statistical methods were used for all main analyses. Due to the relative small samples non-parametric permutation tests were used to achieve maximum power. For comparison of both efficacy variables and baseline variables between the two groups, Fisher’s non-parametric permutation test was used for continuous variables, Fisher’s exact test for dichotomous variables, Mantel-Haenszel chi-square test for ordered categorical variables and chi-square test for non-ordered categorical variables. Last observation carried forward was applied to all missing primary and secondary efficacy variables data after the baseline, but baseline values were not carried forward. Time to healing were described with Kaplan-Meier curves and analysed unadjusted with log-rank test and adjusted with a Cox proportional hazard regression model.

All efficacy analyses were performed on both the intention-to-treat (ITT) population and the per protocol (PP) population. All ITT analyses were based on as-randomized treatment groups. All PP and safety analyses were based on as-treated (actual) treatment groups. PP results are presented in the results section.

All tests were two-tailed and conducted at 5 % significance level, and carried out using SAS® v9.4 (Cary, NC, USA).

## Results

### Study cohort

In all, 40 patients were randomised, 21 to chloramines treatment and 19 to standard care. In the chloramine-treated group, one patient was excluded because he had not reported a recent percutaneous angioplasty, one who made irregular contact and was included in the study at two centres, one because two ulcers coalesced and were not possible to evaluate, and one who missed 6 appointments. One patient was erroneously included in spite of a lower toe blood pressure than 30 mmHg. This violation of the protocol was accepted due to difficulties to measure adequately, since the first toe had recently undergone surgery, and clinically estimated adequate circulation in area of the ulcer, which was not located on the first toe. In the control group, one patient was lost to follow-up after the baseline visit, while one chose to withdraw informed consent. Thus, 17 patients in each group completed the 12 weeks study. Only one patient in each group used a wheelchair (apart from individually tested orthopaedic shoes and inserts), and one patient in each group used so-called Aircasts during parts of the follow-up period.

### Baseline characteristics

The mean ages (and SD, standard deviations) of the participants were 67.5 (SD11.8) and 74.5 (SD 12.3) years in the patients randomized to chloramine treatment and the control group, respectively, with duration of diabetes of more than 20 years (Table 1). The majority were male, non-smoking, with overweight and unsatisfactory glycaemic and blood pressure control, but with generally high physical activity. Most of the participants exhibited sensory neuropathy while retinopathy, as well as a history of coronary heart disease, was present in almost half of the patients. More than half of the patients had had amputation of a foot or one leg, and the majority had a history of foot ulcer. The mean durations of the current ulcers were 1.78 (SD 3.26) and 1.19 (SD 1.69) years, respectively, and the mean ulcer area was 542 (SD 927) mm^2^ in patients randomized to chloramine treatment and 428 (SD 713) mm^2^ in the control group. There were no statistically significant differences between the groups in any of the baseline characteristics, and a sensitivity analysis (linear regression) showed that the distribution of characteristics between the groups was not skewed.

**Table 1:** Baseline characteristics

Variable	Chloramine (*n* = 17)	Standard treatment (*n* = 17)
Age (years)	67.5 (11.8) 69.6 (43.4; 86.1)	74.5 (12.3) 76.9 (47.1; 90.5)
Sex (M/F, *n*)	14/3	11/6
Diabetes duration (years)	21.8 (12.1) 17.5 (7.0; 41.0)	22.6 (13.4) 20.0 (2.0; 52.0)
HbA1c (mmol/mol)	67.0 (15.5) 66.0 (42.0; 94.0)	66.4 (18.3) 66.0 (33.0; 96.0)
BMI (kg/m^2^)	28.1 (3.9) 27.6 (20.6; 35.8)	26.5 (4.3) 26.0 (20.5; 36.0)
Systolic blood pressure (mmHg)	143.3 (22.0) 145.0 (100.0; 177.0)	149.9 (16.1) 150.0 (120.0; 175.0)
Diastolic blood pressure (mmHg)	76.0 (9.8) 75.0 (54.0; 89.0)	70.4 (12.7) 70.0 (50.0; 91.0)
Toe blood pressure (mmHg)	75.0 (30.9) 82.0 (16.0; 123.0)	62.3 (32.9) 52.0 (32.0; 152.0)
Non-smoking status (*n*)	15	14
Alcohol habits (audit)	3.29 (3.22) 3.00 (0.00; 11.00)	2.13 (2.09) 2.00 (0.00; 5.00)
Physical activity (inactive, low, high; *n*)	3/1/12	2/3/11
Previous stroke/TIA (no/yes; *n*)	15/4	17/1
Coronary heart disease (no/yes; *n*)	11/8	11/7
Retinopathy (no/yes; *n*)	9/8	7/11
Neuropathy	1/18	3/15
Amputation (no/yes: *n*)	9/9	6/11
Previous foot ulcer (no/yes; *n*)	3/15	3/15
Duration of current ulcer (years)	1.78 (3.26) 0.46 (0.12; 14.00)	1.19 (1.69) 0.47 (0.07; 5.19)
Ulcer area (mm^2^)	542 (927) 192 (28; 3654)	428 (713) 190 (27; 2928)

For categorical variables n is presented. For continuous variables mean (SD)/median (min; max)/*n* = are presented. All comparisons between the groups: n.s.

### Changes in ulcer size

**Fig. 1:**
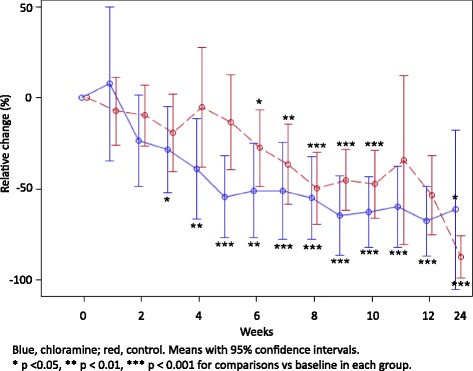
Relative change in ulcer area (%) per treatment group and visit

Figure 1 shows the change in ulcer area in the two treatment groups relative to the baseline value. In the group treated with chloramine, there was a significant reduction in ulcer size after three weeks and after six weeks in the control group. At week five, the relative reduction in ulcer area was statistically significant (*p* = 0.016) between the groups. The absolute change in ulcer area was first statistically significant in the chloramine-treated group after two weeks (*p* = 0.026) and after eight weeks in the control group (*p* = 0.0023), with a statistically significant difference between the groups after five weeks (*p* = 0.024). There was no significant difference between the groups in the primary efficacy variable (absolute change in area of the ulcers from the baseline visit to week 12).

**Fig. 2:**
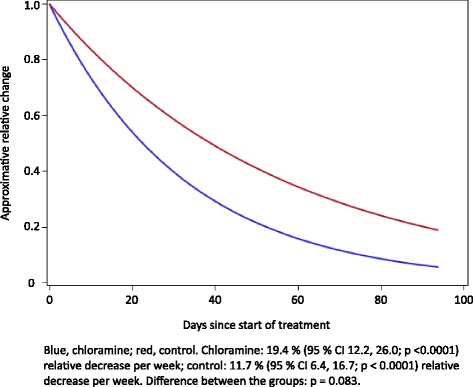
Relative change in wound area as function of time

Figure 2 gives the relative changes in ulcer area as a function of time. The approximate relative decrease per week was 19.4 % (95 % CI 12.2, 26.0; *p* <0.0001) in the chloramine-treated group and 11.7 % (95 % CI 6.4, 16.7; *p* < 0.0001). This difference in relative decrease between the groups did not reach statistical significance (*p* = 0.083).

### Time to healing

**Fig. 3:**
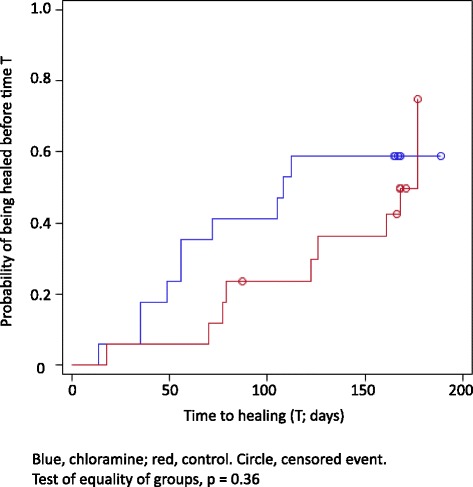
Time to healing

During the early phase of the trial, the number of healed ulcers was numerically higher in the chloramine-treated group (Fig. 3). After nine weeks seven patients had healed in the that group, but only one in the control group producing statistical significance (comparison between groups *p* = 0.039). After 12 weeks two more ulcers had healed in the control group, producing a statistically non-significant difference between the groups. After 24 weeks 10 of the ulcers had healed in the chloramine group and nine in the control group. Figure 3 gives probability of being healed during the course of the full 24 weeks follow-up period.

### Infection, pain, EQ-5D, and modelling

There were only minimal and non-significant changes in HbA1c and CRP during the study. Signs of infection were similar in the two groups throughout the follow-up period. At the baseline visit, signs of infection were present in 13 and 15 of the chloramine and the control groups, respectively, while after 12 weeks 4 and 8 ulcers, respectively, showed such signs. Although there were statistically significant reductions within the groups during the follow-up period, there were no significant differences between the groups. The use of antibiotic treatment was more than 50 % during the 12 weeks follow-up period (not statistically different between the two groups). Pain measured with a visual-analogue scale had improved significantly after 10 weeks in both groups (no difference in pain in the groups). EQ-5D did not show a significant improvement until the 24 weeks visit in the chloramine group (no statistically significant difference between the groups).

There was large heterogeneity in ulcer sizes at baseline, but a sensitivity analysis (linear regression), with all baseline characteristics, including size and location of the ulcer, toe blood pressure, number of follow-up visits or variability in number of days between visits, revealed that none of these variables was shown to affect the decrease in ulcer area during the study (all p–values >0.05).

### Safety

In the chloramine-treated group, the number of pre-specified adverse events was 7 patients with increased pain, 4 with non-study related injuries, and 2 with 50 % increase in wound size (a few patients had more than one adverse event). In the control group, 5 patients experienced increased pain, 3 had non-study related injuries (6 injuries in total), and 2 had 50 % increase in wound size.

There was also 1 fracture in each group (not study-related). In the chloramine-treated group, 5 patients required a vascular procedure or an amputation of a toe (in 1 case 10 days after the baseline visit due to tight sutures after surgery prior to study start, and the other one after 17 weeks). In the control group, 3 patients required a vascular procedure, one of whom had a lower leg amputation (on the side of the foot ulcer) after 17 weeks.

## Discussion

This explorative open randomized controlled trial shows that chloramine can safely be used in the treatment of infected foot ulcers in diabetes. The absolute and relative ulcer area improved, time to healing was shorter, and the number of healed foot ulcers was increased in patients randomised to chloramine treatment during the first two months of the study. These differences, including the primary outcome (absolute change in area of the ulcers from the baseline visit to week 12), were not, however, statistically significant after 12 and 24 weeks, suggesting that the efficaciousness of chloramine-based weekly treatment is predominantly in the early phases of ulcer treatment.

Wound healing has several phases, including haemostasis, inflammation, proliferation and granulation, and remodelling, eventually closing the wound. Patients with diabetes mellitus often show impaired function of the local immune system, and chronic ulcers could stall in the inflammatory phase [23]. The patients participating in this trial were around 70 years of age, with diabetes duration of more than 20 years, a history of micro- and macrovascular complications, and exhibited unsatisfactory risk factor control. The long duration of the ulcers of the patients in the study (mean around 1.5 years), clearly illustrates the multifactorial nature of foot ulcers in diabetes and the difficulties involved in developing methods and improving wound care results [3].

Chloramines are not cytotoxic and have several properties, which could prove useful in the treatment of infected wounds and ulcers, and thus might have contributed to the beneficial effects in this trial [10, 24]. Subjectively, we had the impression of a more rapid transformation from black or yellow necrosis in the ulcers to purulence and red granulation than in the control group. After reaching this phase, the ulcer healing seemed to be similar in the two treatment groups. The ulcers also appeared more easily debrided without damaging the underlying tissue.

This study is limited by its open design, exploratory nature and limited size, as well as the use of different dressings required by the current status of the ulcers. Differences in off-loading might also have been a limitation, but use of a wheelchair was only used in one case in each group, and only one patient in each group used a removable cast walker boot temporarily. Wound care is a dynamic process, requiring appropriate handling and dressings at different stages. The non-adhesive protective dressing used in the chloramine-treated group might have been advantageous in the first phases but a disadvantage later in the healing process. The active dressing to keep a moist environment might similarly have been a disadvantage in the early treatment of the control group but an advantage later on. Furthermore, the methods used to characterize the ulcers could be improved, determining also ulcer volume and in more detail the current stage in the healing process. The groups, however, shared the same clinical characteristics, and attended clinics and were cared for by experienced podiatrists, nurses and physicians. The indicator was current standard of care, including even more frequent visits than usually offered, suggesting that a treatment regimen based on use of chloramine could represent an important addition to wound care.

## Conclusions

Chloramine-based treatment seems to be efficacious, particularly in the early phase of the care of infected diabetic foot ulcers. It is safe and easy to use, and could prove to be a valuable addition in the treatment arsenal, providing non-surgical debridement. The results of this exploratory study will provide the basis for adequately dimensioned forthcoming studies addressing the effects in the healing of wounds and ulcers, not only in patients with diabetes. Mechanistic studies are also needed to study the effects in practice on infection, biofilm, local immune response and the wound-healing cascade.

## Ethics, consent and permissions

Informed consent was obtained from all participants.

## References

[1] Boulton AJ, Vileikyte L, Ragnarson-Tennvall G, Apelqvist J (2005). The global burden of diabetic foot disease. Lancet.

[2] Apelqvist J (2012). Diagnostics and treatment of the diabetic foot. Endocrine.

[3] Game FL, Apelqvist J, Attinger C (2016). Effectiveness of interventions to enhance healing of chronic ulcers of the foot in diabetes: a systematic review. Diabetes Metab Res Rev.

[4] Hunt DL. Diabetes: foot ulcers and amputations. BMJ Clin Evid(Online). 2011;2011:1–44. PMC3275104 21871137

[5] Martin P, Nunan R (2015). Cellular and molecular mechanisms of repair in acute and chronic wound healing. Br J Dermatol.

[6] Bjarnsholt T, Kirketerp-Moller K, Jensen PO (2008). Why chronic wounds will not heal: a novel hypothesis. Wound Repair Regen.

[7] Davis SC, Ricotti C, Cazzaniga A, Welsh E, Eaglstein WH, Mertz PM (2008). Microscopic and physiologic evidence for biofilm-associated wound colonization in vivo. Wound Repair Regen.

[8] James GA, Swogger E, Wolcott R (2008). Biofilms in chronic wounds. Wound Repair Regen.

[9] Almhojd U, Jansson H, Roos-Jansaker A-M, Campus G, Lingstrom P (2015). The Antimicrobial Effect of Sodium Hypochlorite Agents for Intraoral Use.

[10] Gottardi W, Debabov D, Nagl M (2013). N-chloramines, a promising class of well-tolerated topical anti-infectives. Antimicrob Agents Chemother.

[11] Gottardi W, Nagl M (2010). N-chlorotaurine, a natural antiseptic with outstanding tolerability. J Antimicrob Chemother.

[12] Tawakoli PN, Ragnarsson KT, Rechenberg DK, Mohn D, Zehnder M. Effects of endodontic irrigants on biofilm matrix polysaccharides. Int Endod J. 2015. doi: 10.1111/iej.12604. 10.1111/iej.12604 26705856

[13] Hannig M (1999). Effect of Carisolv solution on sound, demineralized and denatured dentin--an ultrastructural investigation. Clin Oral Investig.

[14] Pai VS, Nadig RR, Jagadeesh T, Usha G, Karthik J, Sridhara K (2009). Chemical analysis of dentin surfaces after Carisolv treatment. J Conserv Dent.

[15] Roos-Jansaker AM, Almhojd US, Jansson H. Treatment of peri-implantitis: clinical outcome of chloramine as an adjunctive to non-surgical therapy, a randomized clinical trial. Clin Oral Implants Res. 2015. doi: 10.1111/clr.12612. 10.1111/clr.12612 26013241

[16] Wennerberg A, Sawase T, Kultje C (1999). The influence of Carisolv on enamel and dentine surface topography. Eur J Oral Sci.

[17] Lipsky BA, Peters EJ, Senneville E (2012). Expert opinion on the management of infections in the diabetic foot. Diabetes Metab Res Rev..

[18] Johansson KE, Marklund BR, Fowelin JH (2002). Evaluation of a new screening method for detecting peripheral arterial disease in a primary health care population of patients with diabetes mellitus. Diabet Med.

[19] Baker RW (1947). Studies on the reaction between sodium hypochlorite and proteins; physico-chemical study of the course of the reaction. Biochem J.

[20] Hawkins CL, Pattison DI, Davies MJ (2003). Hypochlorite-induced oxidation of amino acids, peptides and proteins. Amino Acids.

[21] Game FL, Hinchliffe RJ, Apelqvist J (2012). Specific guidelines on wound and wound-bed management 2011. Diabetes Metab Res Rev..

[22] Kajagar BM, Godhi AS, Pandit A, Khatri S (2012). Efficacy of low level laser therapy on wound healing in patients with chronic diabetic foot ulcers-a randomised control trial. Indian J Surg.

[23] Portou MJ, Baker D, Abraham D, Tsui J (2015). The innate immune system, toll-like receptors and dermal wound healing: A review. Vascul Pharmacol..

[24] Thomas EL, Jefferson MM, Grisham MB (1982). Myeloperoxidase-catalyzed incorporation of amines into proteins: role of hypochlorous acid and dichloramines. Biochemistry.

